# An integrated analysis of the rice transcriptome and lipidome reveals lipid metabolism plays a central role in rice cold tolerance

**DOI:** 10.1186/s12870-022-03468-1

**Published:** 2022-03-02

**Authors:** Hualong Liu, Wei Xin, Yinglin Wang, Dezhuang Zhang, Jingguo Wang, Hongliang Zheng, Luomiao Yang, Shoujun Nie, Detang Zou

**Affiliations:** 1 Key Laboratory of Germplasm Enhancement, Physiology and Ecology of Food Crops in Cold Region, Ministry of Education, Northeast Agricultural University, 150030 Harbin, China; 2 Innovation Center, Suihua Branch of Heilongjiang Academy of Agricultural Science, 152052 Suihua, China

**Keywords:** Rice, Transcriptome, Lipidomic, Cold stress

## Abstract

**Background:**

Rice (*Oryza sativa* L.) is one of the most widely grown food crops, and its yield and quality are particularly important for a warm-saturated diet. Cold stress restricts rice growth, development, and yield; however, the specific mechanism of cold tolerance in rice remains unknown.

**Results:**

The analysis of leaf physiological and photosynthetic characteristics showed that the two rice varieties were significantly affected by cold stress, but the cold-tolerant variety KY131 had more stable physiological characteristics, maintaining relatively good photosynthetic capacity. To better explore the transcriptional regulation mechanism and biological basis of rice response to cold stress, a comprehensive analysis of the rice transcriptome and lipidome under low temperature and control temperature conditions was carried out. The transcriptomic analysis revealed that lipid metabolism, including membrane lipid and fatty acid metabolism, may be an important factor in rice cold tolerance, and 397 lipid metabolism related genes have been identified. Lipidomics data confirmed the importance of membrane lipid remodeling and fatty acid unsaturation for rice adaptation to cold stress. This indicates that the changes in the fluidity and integrity of the photosynthetic membrane under cold stress lead to the reduction of photosynthetic capacity, which could be relieved by increased levels of monogalactosyldiacylglycerol that mainly caused by markedly increased expression of levels of 1,2-diacylglycerol 3-beta-galactosyltransferase (MGD). The upregulation of phosphatidate phosphatase (PAP2) inhibited the excessive accumulation of phosphatidate (PA) to produce more phosphatidylcholine (PC), phosphatidylethanolamine (PE), and phosphatidylglycerol (PG), thereby preventing of membrane phase transition under cold stress. In addition, fatty acid β-oxidation is worth further study in rice cold tolerance. Finally, we constructed a metabolic model for the regulatory mechanism of cold tolerance in rice, in which the advanced lipid metabolism system plays a central role.

**Conclusions:**

Lipidome analysis showed that membrane lipid composition and unsaturation were significantly affected, especially phospholipids and galactolipids. Our study provides new information to further understand the response of rice to cold stress.

**Supplementary Information:**

The online version contains supplementary material available at 10.1186/s12870-022-03468-1.

## Background

With population growth, the demand for food is increasing daily. Rice (Oryza sativa L.) is one of the most widely grown food crops, and its yield and quality are particularly important for a warm-saturated diet [1]. The rice planting area in the world is approximately 140 million hectares, in particular, more than 15 million hectares are threatened by different degrees of low-temperature damage every year. Chilling damage to rice has led to a reduction in yield and quality, which greatly affects food security, and owing to latitude, this problem is more prominent in China [2, 3]. The phenomenon of “spring cold” before and after transplanting rice occurs occasionally. It may delay the growth process of rice, delay the rice plant turning green, reduce the number of tillers, and seriously cause the death of rice seedlings to varying degrees. Some rice varieties with poor cold tolerance die completely, and farmers will need to replant the seedlings. Therefore, cold tolerance of rice germplasm is an unavoidable challenge, for which research can have an important scientific significance in improving cold tolerance of rice and considerably improving the economic value of people’s quality of life.

Presently, it is widely considered that the model of plant cold tolerance signal transduction mechanism is initiated when cold stress is first detected by plasma membrane proteins (such as calcium channels or related proteins), leading to calcium influx and then to changes in membrane fluidity and cytoskeletal rearrangement. Then, calcium-responsive protein kinases, including calcium-dependent protein kinases (CDPKs), calcineurin B-like interacting protein kinases (CIPKs), and calcium-regulated receptor-like kinases (CRLKs), may mediate calcium signals to activate the mitogen-activated protein kinase (MAPK) cascade, which can regulate cold tolerance-related gene expression and improve cold stress tolerance [4–6]. The expression of *ZmMKK1* in maize is induced by various stresses and exogenous signaling molecules. During cold stress, the content of osmotic regulators, antioxidant enzyme activities, and expression of stress-related genes in tobacco plants overexpressing *ZmMKK1* are significantly increased, thereby increasing the cold tolerance of genetically modified tobacco [7]. Concurrently, these transcriptome changes are regulated by a complex network of transcription factors (TFs) and other regulatory proteins and RNAs. TFs act as transcriptional activators or repressors and play a central role in the regulation of development, metabolic processes, and biotic and abiotic stresses. Several studies have shown that transcription factors also play an important role in the adaptation of rice to low-temperature stress [8–11].

Previous studies have shown that the regulation mechanism of biofilm fluidity is one of the main mechanisms by which plants adapt to temperature changes, which is affected by the distribution ratio of various lipids on the membrane and the degree of unsaturation [12]. One of the newest research discoveries in the field of cold signal transduction is the formation of lipid second messengers, phosphatidic acid (PA), which accumulates in cells in suspension culture within minutes under cold stress [13, 14]. During cold-induced signaling, PA responds to cold stress mainly through two pathways: one is a direct product of phospholipase D (PLD), which hydrolyzes structural phospholipids such as phosphatidylcholine (PC) and phosphatidylethanolamine (PE), and the other is a secondary product of the phospholipase C (PLC) pathway, which first polyphosphoinositide (PPI) is hydrolyzed to diacylglycerol (DG), which is subsequently phosphorylated to PA with diacylglycerol kinase (DGK) [15, 16]. Furthermore, PA is the synthetic precursor to all phosphoglycerolipids, turnoveris crucialin, galactolipids, and triacylglycerols (TGs), which determines lipid metabolic flux and membrane composition [17]. Membrane lipid remodeling is another important factor in plant cold tolerance, which has been demonstrated in several *algaes*, *Arabidopsis*, and several crops, with specific variations among different plants [18–20]. Previous studies in non-plant systems have shown that in addition to PA, cold stress also induces responses to various lipid signals, such as phosphatidylinositol (PI), lysophospholipids, and sphingolipids [21]. However, how these specific lipid species are produced and further affect plant cold tolerance under cold stress is unclear.

Biomembranes are the barrier for cells and organelles to contact the outside environment and are also the most sensitive to temperature changes. Under low temperature stress, the increase in membrane permeability and decrease in fluidity caused by the phase transition of membrane lipids are the root causes of cold damage to plants [22]. To resist low-temperature damage, plants have formed a complex mechanism that adapts to temperature changes through membrane lipid remodeling and adjustment of fatty acid unsaturation during long-term evolution [12, 18, 20]. In this study, cold-tolerant rice varieties Kongyu 131 (KY 131) and cold-sensitive rice varieties Dongnong 422 (DN 422) were used as experimental materials. Using lipid detection technology, the membrane lipid composition and fatty acid (FA) content in the leaves of rice seedlings were determined under low-temperature stress. This was combined with transcriptome data to analyze the lipid metabolism pathway. Clarifying the regulation mode of lipid metabolism in rice under low temperature stress, will establish the basis for analyzing the regulation mechanism of lipid metabolism and improve rice cold tolerance.

## Results

### Responses of physiological and photosynthetic characteristics of rice during cold stress

**Fig. 1:**
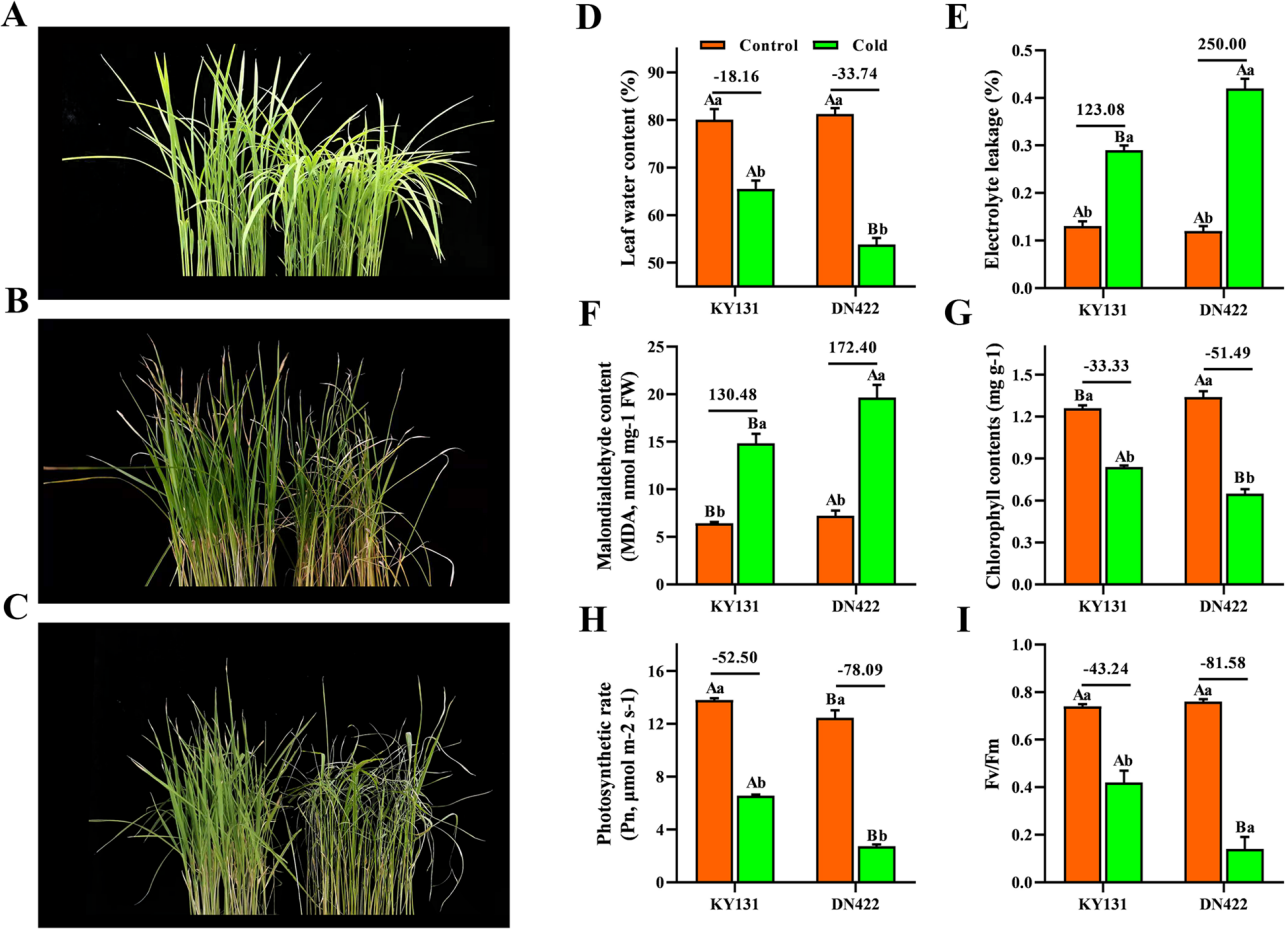
Leaf phenotypes, physiological and photosynthetic characteristics responses of rice during cold stress. **A** phenotypes of KY 131 and DN 422 under control conditions; **B** phenotypes of KY 131 and DN 422 under cold conditions; **C** phenotypes of KY 131 and DN 422 under recovery conditions; **D** leaf water content of KY 131 and DN 422 under cold stress; **E** electrolyte leakage of KY 131 and DN 422 under cold stress; **F** malondialdehyde of KY 131 and DN 422 under cold stress; **G** the chlorophyll contents of KY 131 and DN 422 under cold stress; **H** photosynthetic rate of KY 131 and DN 422 under cold stress; **I** the maximum photochemical efficiency of PSII of KY 131 and DN 422 under cold stress. Different lowercase and uppercase letters represent significant differences among treatments and cultivars, respectively. (as evaluated with ANOVA with Fisher’s LSD, *p* < 0.05. *n*=5)

After nine days of cold treatment **(**Fig. 1 A, B**)**, the two varieties showed withered leaf tips and curled leaves. After five days of recovery treatment **(**Fig. 1 C**)**, KY131 resumed normal growth except for withered yellow leaf tips, while DN422 showed dry leaves. Based on the cold response of the two varieties, we selected samples processed for nine days to study the relevant physiological and molecular mechanisms. Under cold conditions, the leaf water content in KY 131 and DN 422 decreased, while the water content in KY 131 was significantly higher than that in 422 under cold conditions. The MDA content in KY 131 and DN 422 increased by 130.48% and 172.40%, respectively. The EL in KY 131 and DN 422 increased by 123.08% and 250.00%, respectively. The photosynthetic capacity of KY 131 and DN 422 seedlings after nine days of cold stress was estimated by the Chl content, net photosynthetic rate (Pn), and Fv/Fm, respectively. Under cold conditions, the Chl content, Pn, and Fv/Fm were both decreased in the two rice cultivars, while the levels in KY 131 were significantly higher than those in DN 422 (Fig. 1 D-I). These physiological responses indicated that KY 131 was relatively stable under cold conditions.

### Cold response transcriptome differences in rice cultivars

**Fig. 2:**
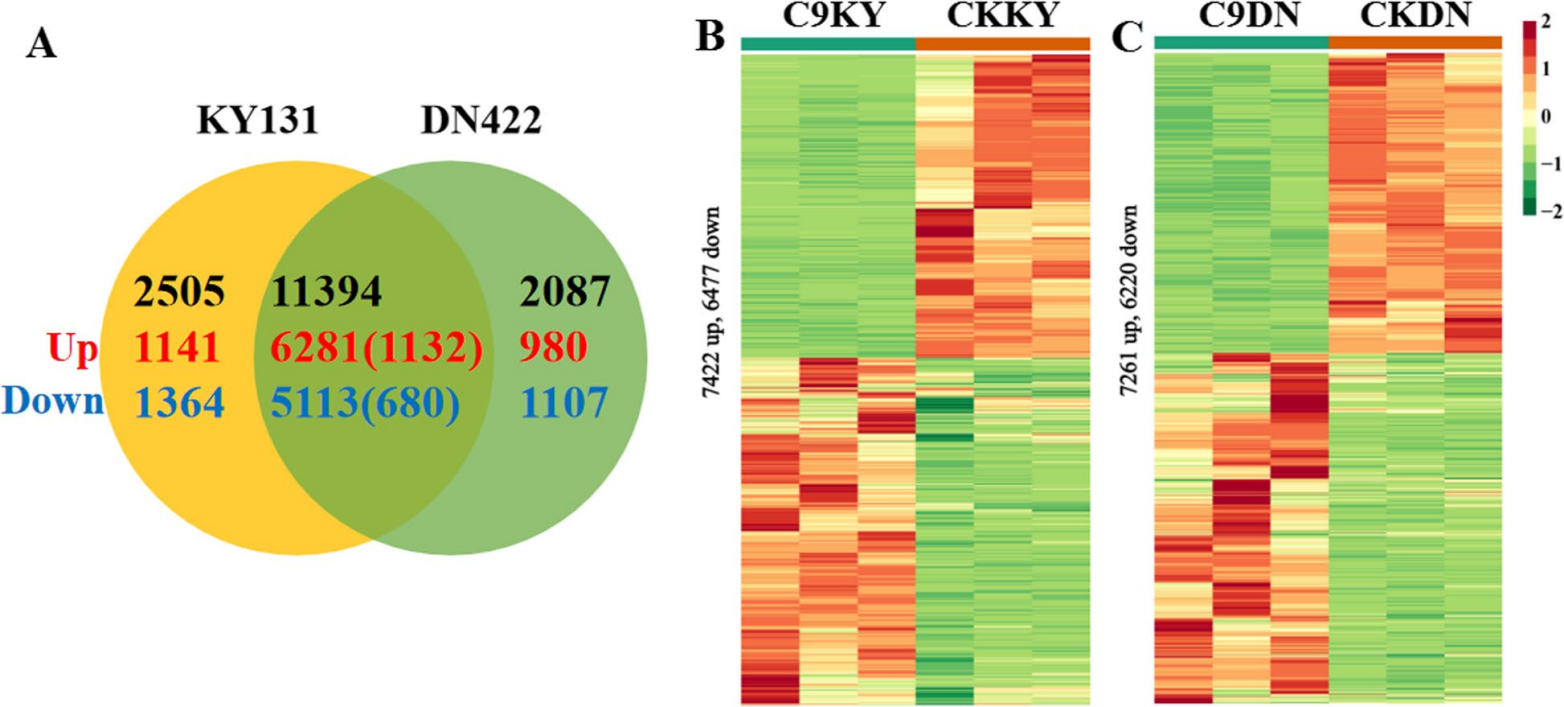
Differentially expressed transcripts in KY 131 and DN 422 under cold conditions. **A** Venn diagram showing the number of transcripts detected as up and down-regulated (*P* < 0.01) in KY 131 and DN 422 under cold conditions; **B** Heat maps showing differentially expressed transcripts in KY 131 under control and cold conditions; **C** Heat maps showing differentially expressed transcripts in DN 422 under control and cold conditions

There were significant differences in the physiological and photosynthetic characteristics between KY 131 and DN 422. To understand the regulatory mechanisms of cold stress and explore new regulators, cold-responsive transcriptome changes in the leaves of KY 131 and DN 422 were studied using RNA-seq. Differentially expressed genes (DEGs) were examined using the R package DEseq2 to quantify and analyze all the control and treatment conditions. Over the nine days of cold exposure, 15,986 genes were significantly differentially expressed under cold stress, and of these, 11,394 genes were commonly differentially expressed in both varieties, while 2505 and 2087 genes were specifically expressed in KY131 and DN422, respectively (Tables S1, 2). Moreover, the differential expression level of 1812 genes of 11,394 common differentially expressed genes was significantly higher in KY131 than in DN422 (FC(KY131/DN422) ≥ 1.5), which comprised the “1812 genes of 11394” and with the 2505 differentially expressed genes only in KY131 and may play a central role in rice cold tolerance (Fig. 2).

### The Gene Ontology (GO) and Kyoto Encyclopedia of Genes and Genomes (KEGG) enrichment analysis

**Fig. 3:**
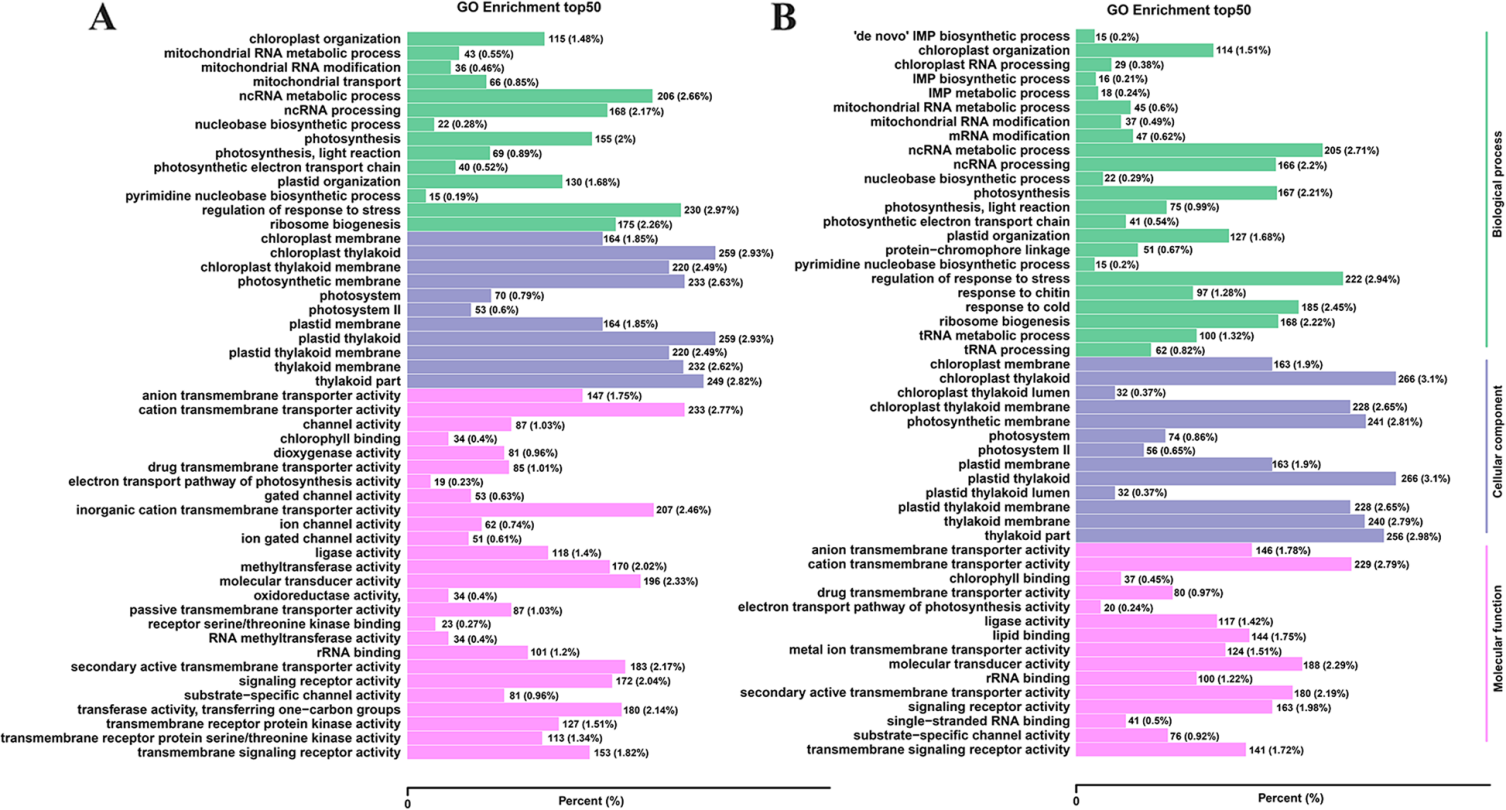
The Gene Ontology (GO) enrichment analysis under cold conditions. **A** the top 50 significantly rich GO terms in KY 131 under cold conditions; **B** the top 50 significantly rich GO terms in DN 422 under cold conditions

To further determine the functions of the DEGs and the related biological processes they participate in, GO and KEGG enrichment analyses were conducted. The GO database enrichment analysis was separated into three ontologies: ‘cellular component,’ ‘molecular function,’ and ‘biological process.’ A total of 51 and 53 GO terms were significantly different in the CKKY vs. C9KY and CKDN vs. C9DN comparisons, respectively (Tables S3, 4). We selected the 50 GO terms with the lowest false discovery rate (FDR) in the enrichment analysis results and drew a column chart of enrichment items, as shown in Fig. 3. The results of GO enrichment hierarchical analysis revealed that within the cellular component the enriched GO terms in both KY 131 and DN 422 were ‘thylakoid part’, ‘photosynthetic membrane’, ‘thylakoid membrane’, ‘chloroplast thylakoid’, and ‘plastid thylakoid’. Within the molecular function the enriched GO terms in both KY 131 and DN 422 were ‘chlorophyll binding’, ‘drug transmembrane transporter activity’, ‘signaling receptor activity’, and ‘molecular transducer activity’, and the specific term enrichment for KY 131 included ‘transmembrane signaling receptor activity’. Within the biological process the enriched GO terms in both KY 131 and DN 422 were ‘photosynthesis’, ‘ncRNA processing’, ‘chloroplast organization’, ‘ncRNA metabolic process’, and ‘plastid organization’, and the specific term enrichment for KY 131 included ‘mitochondrial transport’, ‘ribosome biogenesis’, and ‘ribonucleoprotein complex biogenesis’. The GO enrichment analysis results showed that the function of the photosynthetic membrane, chloroplast membrane, thylakoid membrane, and other biomembranes, as well as transmembrane substances and signal conduction processes, are significantly affected by low temperature stress, indicating that the stability of biofilms under low temperature stress is closely related to the low-temperature tolerance of rice.

**Fig. 4:**
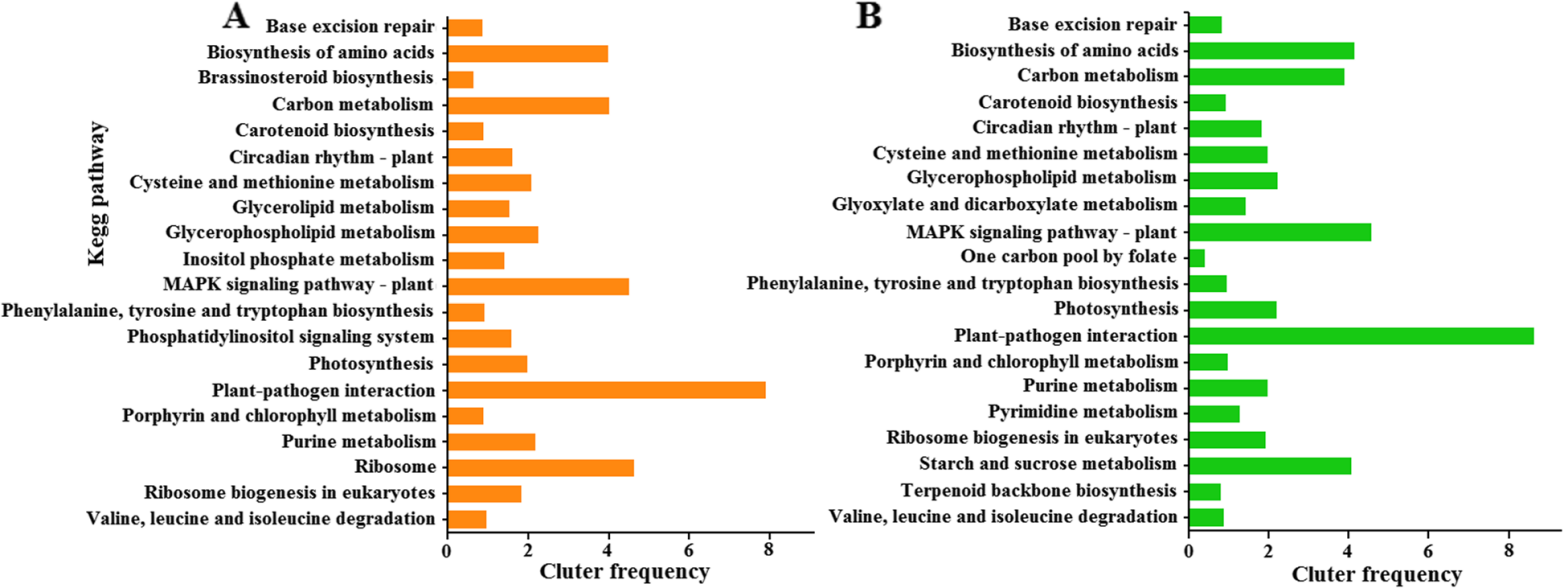
The Kyoto Encyclopedia of Genes and Genomes (KEGG) enrichment analysis under cold conditions. **A** the top 20 significantly rich pathways in KY 131 under cold conditions; **B** the top 20 significantly rich pathways in DN 422 under cold conditions

The KEGG pathway enrichment analysis was conducted to determine the biological pathways involved in cold stress (Tables S5, 6). The top 20 pathways were significantly enriched in the CKKY vs. C9KY and CKDN vs. C9DN groups (Fig. 4). These metabolic pathways were mainly enriched in ‘carbohydrate metabolism,’ ‘amino acid metabolism,’ ‘lipid metabolism’, MAPK signaling pathway, plant-pathogen interaction, and stress-resistance-related metabolic pathways (MAPK signaling pathway, plant-pathogen interaction). The MAPK signaling pathway plays an important role in plants adapted to cold stress. There were 188 DEGs involved in the MAPK signaling pathway in CKKY vs. C9KY, of which only 184 were also DEGs in CKDN vs. C9DN. In the MAPK signaling pathway in CKKY vs. C9KY, 19 genes were specifically upregulated, and 11 were specifically downregulated (Table S7).

### Cold response transcription factors differences in rice cultivars

**Fig. 5:**
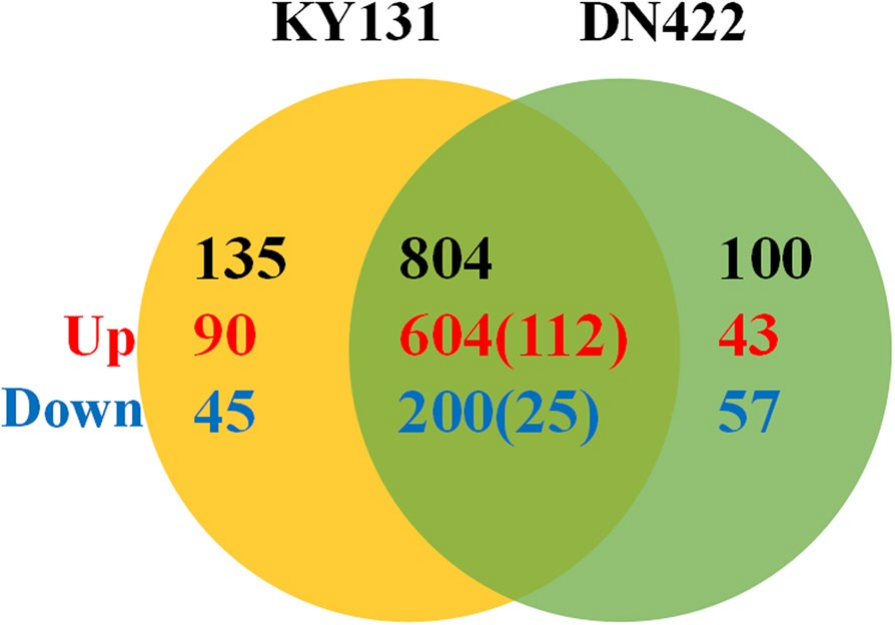
Transcription factors (TFs) differentially expressed in KY 131 and DN 422 under cold conditions

**Fig. 6:**
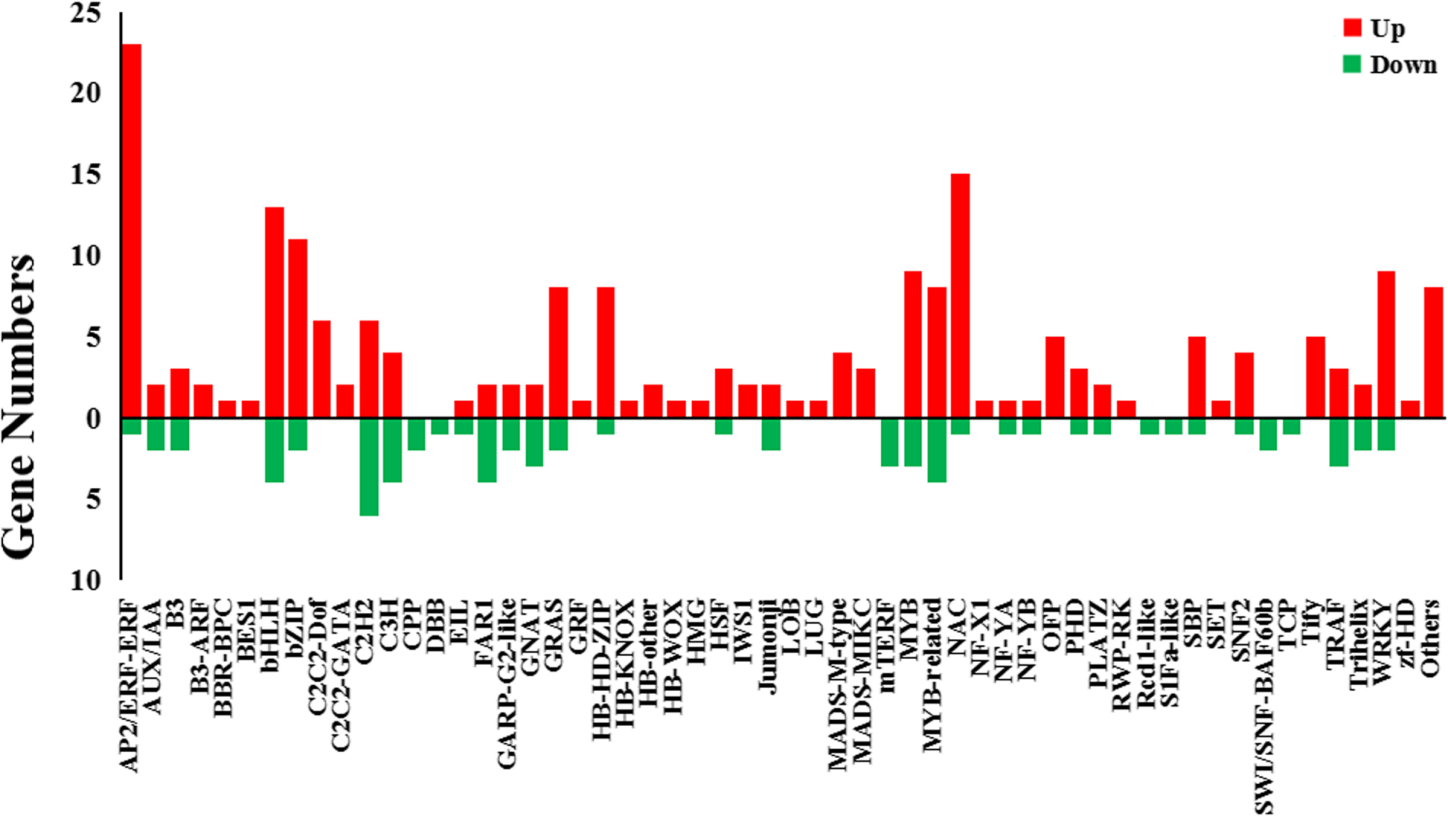
The number of differentially expressed transcriptional factors in the “cold-tolerant transcription factor (TF) set” under cold stress. The X-axis indicates various transcription factor families; Y-axis indicates the number of differentially expressed transcriptional factors. Red bars indicate upregulated; green bars indicate downregulated

Transcription factors (TFs) play important roles in plant growth and response to environmental changes by mediating gene expression. As shown in Fig. 5, under cold stess conditions, 939 TFs were differentially expressed in KY131, and 904 TFs were differentially expressed in DN422. Among them, 135 TFs were specifically expressed in cold-tolerant cultivar KY131, and the expression changes of 137 TFs in KY131 were significantly higher than those in DN422 (FC(KY131/DN422) ≥ 1.5), which genes may play a central role in cold tolerance in rice (Fig. 6). These transcription factors cover a total of 55 transcription factor families, among which AP2/ERF-ERF, bHLH, bZIP, MYB and NACZ transcription factor family members are more, and most of them are up-regulated under cold stress. These results transcriptional regulation plays an important role in rice adaptation to temperature changes and is an important part of rice response to cold stress.

### Lipid metabolism-related genes

**Fig. 7:**
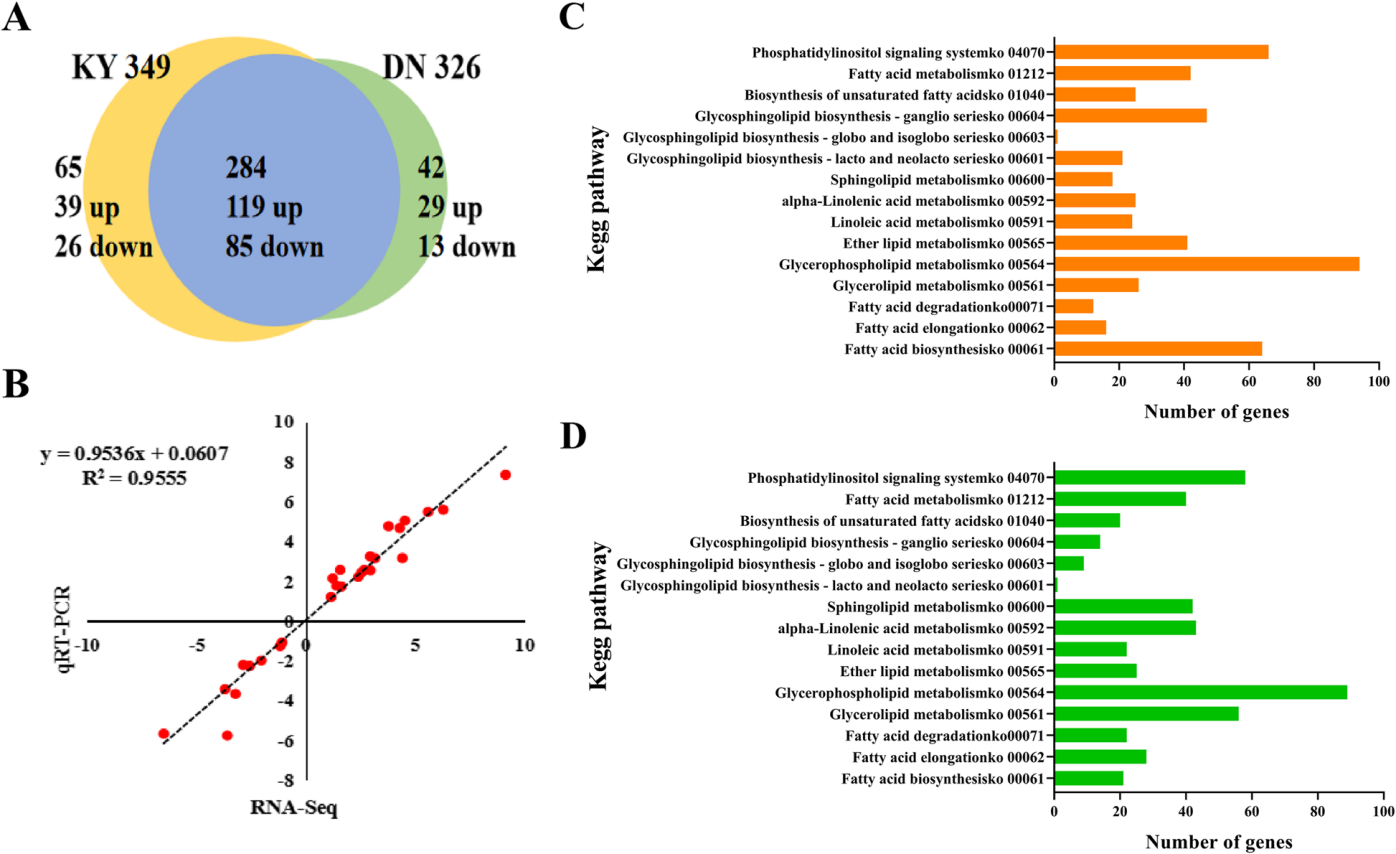
Identification and analyses of DEGs involved in lipid metabolic pathways during the cold stress in KY 131 and DN 422. **A** Venn diagram of lipid metabolism-related genes as up and down-regulated (*P* < 0.01) in KY 131 and DN 422 under cold conditions; **B** Quantitative real-time PCR (qRT-PCR) assay was carried out for lipid metabolism-related DEGs in KY 131 and DN 422; **C** The transcriptional changes involved in 14 lipid metabolic pathways in KY 131 under cold stress; **D** The transcriptional changes involved in 14 lipid metabolic pathways in DN 422 under cold stress

Transcriptional profiling of two rice cultivars indicated that lipid metabolism may play a central role in rice cold tolerance; therefore, we further focused our analyses on the changes in transcription activity of a compiled list of 391 DEGs involved in lipid metabolism. Under cold conditions, 349 DEGs were detected in KY 131, with 238 upregulated and 111 downregulated; 326 DEGs were detected in DN 422, with 228 upregulated and 98 downregulated and of these, 284 DEGs were identified in both varieties, with 199 up-regulated and 85 down-regulated in both varieties, suggesting that lipid metabolism pathways showed significant changes in adaptation to low temperature stress (Fig. 7a). We randomly selected 27 DEGs of lipid metabolism for qRT-PCR in the two varieties. Correlation analysis showed that the qRT-PCR results were highly correlated with the RNA-seq results, with a Pearson coefficient of 0.96 (Fig. 7b). These results showed that the RNA-Seq data were reliable. The KEGG enrichment analysis showed that DEGs were enriched in 14 metabolic pathways including “Fatty acid biosynthesis,” “Fatty acid elongation,” and “Fatty acid degradation” in both two varieties (Fig. 7c, d).

### Quantitative changes in lipid composition induced by cold stress

To investigate whether the differential expression of lipid metabolism genes was reflected in differential lipid abundances, the lipidomes of KY 131 and DN 422 were compared quantitatively under cold stress. In our study, a total of 14 lipid headgroup classes and 218 molecular lipid species were detected (Table 1, S8). The total lipid contents of KY 131 and DN 422 were 13061.27 and 14709.55 nmol mg^−1^ FW under control conditions and were 15932.01 and 14526.62 nmol mg^−1^ FW under cold conditions. Galactolipids, including monogalactosyldiacylglycerol (MGDG), digalactosyldiacylglycerol (DGDG) and sulfoquinovosyldiacylglycerol (SQDG), are the main constituents of chloroplast membranes and photosynthetic complexes, accounting for approximately 66% of the total lipids. The MGDG content in DN 422 was significantly decreased, while in KY 131 it was significantly increased, which was mainly caused by the significant change in MGDG 34:3 and 36:6. The higher MGDG content can inhibit the reduction of chloroplast membrane lipids and maintain the integrity of the chloroplast membrane to a certain extent, thereby inhibiting a significant reduction in photosynthesis.

**Table 1:** The changes of lipid components (nmol g−1 FW) after cold treatments in KY 131 and DN 422. Data are the means ± SD. Means denoted by the same letter do not differ signifificantly according to Tukey's test (p < 0.05)

Lipid class	KY		DN		Relative change (RC)	
	Control	Cold	Control	Cold	KY	DN
MGDG	5084.35±236.46b	6796.77±189.52a	6328.62±534.29a	5249.45±259.11b	33.68	-17.05
DGDG	2256.06±252.23b	2926.05±145.14a	2623.1±252.67a	2894.27±4.65a	29.7	10.34
SQDG	1121.07±126.96a	942.53±79.75a	1191.25±14.00a	1080.82±48.24a	-15.93	-9.27
PA	69.44±8.18a	52.95±5.79a	52.89±1.11a	61.25±5.99a	-23.75	15.81
PC	309.01±29.72b	448.79±32.48a	364.89±35.2a	319.34±17.01a	45.23	-12.48
PE	469.4±35.85b	591.79±34.2a	487.52±29.78a	444.07±24.58a	26.07	-8.91
PG	1668.29±89.71a	1777.14±17.5a	1740.37±104.9a	1685.85±47.56b	6.52	-3.13
PI	626.04±21.41b	729.07±22.99a	440.92±41.57b	528.48±66.77a	16.46	19.86
PMeOH	18.92±1.06a	18.36±0.98a	21.99±0.68a	21±1.59a	-2.96	-4.5
LPE	14.97±1.09b	18.64±1.64a	26.03±2.58b	41.83±3.00a	24.52	60.7
LPC	17.24±1.39a	16.38±1.19a	23.05±2.42a	23.8±0.36a	-4.99	3.25
DG	856.76±29.89b	1264.52±107.51a	1080.69±92.26b	1766.78±26.59a	47.59	63.49
TG	171.88±4.30a	88.54±6.00b	87.91±7.84b	133±2.28a	-48.49	51.29
FA	377.86±21.73a	260.47±9.89	240.32±19.19b	276.68±2.19a	-31.07	15.13
Total	13061.27±340.00b	15932.01±357.56a	14709.54±636.42a	14526.62±249.47a	21.98	-1.24

Phospholipids are important structural lipids that make up cell membranes. The content of phospholipids in rice leaves accounts for about 21-24% of the total lipid content and of these, PG, PC, PE, and phosphatidylinositol (PI) were the most abundant, and PC, PE, and PI were significantly increased in KY 131, with the PC 34:3, 36:5, and C36:6 species contributing the most to the increase in PC, and the PE 34:3 and 36:5 species contributing more to the increase in PE. In this study, two lyso-phospholipids were detected, including lyso-phosphatidylcholine (LPC) and lyso-phosphatidylethanolamine (LPE), which were significantly increased in KY 131 and DN 422 under cold conditions.

**Fig. 8:**
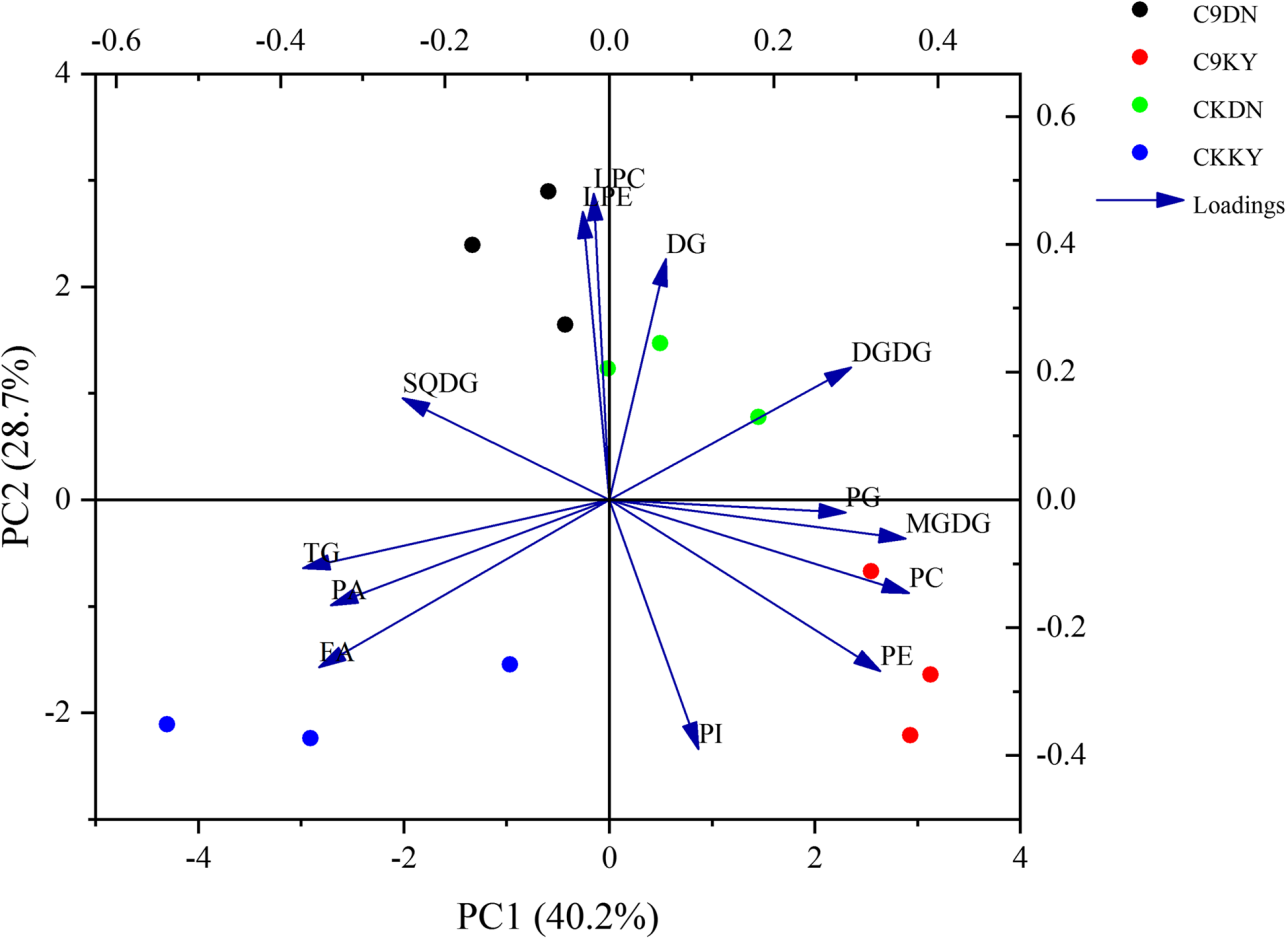
The principal component analysis (PCA) of lipid components determined on rice cultivars when grown with control and cold conditions

In addition to membrane lipids, small amounts of the intermediate lipid DG and the storage lipid triacylglycerol (TG) were also detected. There were 21 DG molecular species in rice leaves and the DG content increased in both varieties under cold conditions. The contents of DG with acyl chains of DG 36:5 and 36:6 were the major contributors to the increased DG. The TG content was significantly reduced in KY 131, and significantly increased in DN 422, under cold conditions, of which TG 52:4, 52:5, 52:6, 54:5, 54:6, 54:7, 54:8, and 54:9 were the main factors causing this result. An increased DG-TG ratio was observed in KY 131, which indicates that the degradation rate of TG was increased in KY 131 under cold conditions. Cold treatment was able to modulate the accumulation of lipid composition in rice. As such, the cold and control treatment were clearly segregated in distinct quadrants of the biplot based on lipid molecular species composition and accumulation. This segregation was based on principal component analysis (PCA), where 68.9% of the variability observed in rice lipids could be explained by both PC1 and PC2. These results showed that MGDG, PG, PC, PE, LPC, and LPE are the main factors affecting the difference in cold tolerance between KY 131 and DN 422 (Fig. 8).

### Changes in the degree of membrane lipid unsaturation induced by cold stress

The above analysis results show that under cold stress, most lipid molecules in rice leaves will change to a certain extent, and there are obvious differences among varieties with different cold tolerance. Changes in the content of lipid molecular species can directly affect the fatty acid groups attached to them, changing their saturation, which in turn affects the unsaturation of membrane lipids. Double bond index (DBI) is the average number of fatty acid double bonds in membrane lipid molecules, and is an important indicator of the total unsaturation of lipids. As shown in Table 2, under cold stress, the DBI of each membrane lipid component in the two rice cultivars changed significantly. The total membrane lipid DBI in KY 131 increased by 26.70%, while the total membrane lipid DBI in DN 422 decreased by 1.53%, indicating that cold-tolerant rice varieties can adapt to cold stress by increasing the membrane lipid unsaturation level. Among the membrane lipid components, MGDG has the highest DBI, which is the main reason for the difference in total membrane lipid unsaturation between KY131 and DN422 under cold stress. In addition, DG, PC, TG, FA, and LPC showed larger RC in DBI ranging from 33.39 to 74.71%, which was another major factor for the increase of total membrane lipid unsaturation.

**Table 2:** Double-bond index (DBI) of membrane lipids after cold treatments in KY 131 and DN 422. DBI = (Ʃ [N × mol % lipid])/100. Data are the means ± SD. Means denoted by the same letter do not differ signifificantly according to Tukey's test (*p* < 0.05)

Lipid class	KY		DN		Relative change (RC)	
	Control	Cold	Control	Cold	KY	DN
MGDG	289.28±13.78b	386.3±11.51a	358.19±30.8a	298.79±14.82b	33.54	-16.58
DGDG	115.62±13b	150.36±6.82a	134.22±12.54b	147.78±1.47a	30.05	10.1
SQDG	49.32±5.57a	41.18±3.19a	49.1±1.09a	45.8±1.44b	-16.5	-6.72
PA	2.26±0.24a	1.8±0.2b	1.75±0.07b	1.97±0.17a	-20.35	12.57
PC	8.74±0.87b	14.04±0.94a	10.55±1.1a	9.96±0.53a	60.64	-5.59
PE	16.45±1.25b	21.86±1.52a	17.33±1.1a	15.88±0.82a	32.89	-8.37
PG	34.03±3.28b	38.18±0.3a	34.32±2.32a	35.66±0.92a	12.2	3.9
PI	5.45±1a	5.66±0.65a	3.85±0.41a	4.2±0.33a	3.85	9.09
PMeOH	0.55±0.02a	0.55±0.03a	0.65±0.02a	0.64±0.04a	0	-1.54
LPE	0.24±0.03a	0.29±0.02a	0.48±0.05b	0.64±0.05a	20.83	33.33
LPC	0.27±0.02a	0.23±0.02a	0.37±0.04a	0.33±0.01a	-14.81	-10.81
DG	35.67±0.81b	58.63±5.45a	48.16±4.44b	84.13±2.05a	64.37	74.69
TG	10.31±0.31a	5.12±0.39b	5.33±0.52b	8.24±0.15a	-50.34	54.6
FA	6.98±0.9a	4.53±0.15b	3.68±0.27a	3.72±0.19a	-35.1	1.09
Total	575.17±20.72b	728.72±18.79a	667.97±35.63a	657.74±13.14a	26.7	-1.53

### Changes in free fatty acid components induced by cold stress

Sixteen FA components were detected in the rice leaves (Table 3). Although the main FA classes were similar, there was great diversity in their contents between the two cultivars under cold stress. The total fatty acid (TFA) content decreased by 31.06% and increased by 15.13% in KY 131 and DN 422. Simultaneously, the content of saturated fatty acids (SFAs) was decreased in KY 131, but increased in DN 422. The content of unsaturated fatty acids (UFAs) was decreased in KY 131, but was almost unchanged in DN 422. In general, KY 131 accumulated more UFAs (161.53 nmol mg^−1^ FW) than DN 422 (138.47 nmol mg^−1^ FW), and exhibited the genetic characteristic of having more UFAs during the adaptation to low temperature. The main reason for this finding may be that KY 131 had a higher FA 18:3 content under cold conditions. Additionally, under cold conditions, the ratio of UFA to SFA was decreased in both varieties, and the ratio of UFA to SFA in KY 131 was higher than that in DN 422 under both conditions, suggesting that higher levels of unsaturated fatty acids are beneficial for rice to adapt to low temperatures.

**Table 3:** The changes of fatty acid components (nmol g−1 FW) after cold treatments in KY 131 and DN 422. Data are the means ± SD. Means denoted by the same letter do not differ signifificantly according to Tukey's test (*p* < 0.05)

Fatty acid	KY		DN		Relative change (RC)	
	Control	Cold	Control	Cold	KY	DN
FA 14:0	1.67 ± 0.20 a	1.32 ± 0.07 b	1.19 ± 0.11 b	1.41 ± 0.05 a	-20.73	18.69
FA 16:0	73.28 ± 6.19 a	53.90 ± 4.99 b	62.53 ± 5.90 b	83.45 ± 3.99 a	-26.45	33.46
FA 17:0	2.42 ± 0.25 a	1.94 ± 0.16 b	2.05 ± 0.19 b	2.60 ± 0.11 a	-19.77	27.24
FA 18:0	31.24 ± 1.66 a	24.10 ± 3.54 b	21.57 ± 3.24 b	29.22 ± 4.34 a	-22.85	35.45
FA 18:1	11.90 ± 0.89 a	7.09 ± 0.72 b	10.82 ± 1.19 a	10.71 ± 0.49 a	-40.42	-1.03
FA 18:2	28.20 ± 5.40 a	15.65 ± 0.69 b	19.41 ± 2.34 a	20.01 ± 1.00 a	-44.51	3.07
FA 18:3	209.34 ± 30.49 a	137.83 ± 5.04 b	105.54 ± 6.86 a	106.51 ± 5.67 a	-34.16	0.91
FA 19:1	0.27 ± 0.03 a	0.24 ± 0.01 a	0.23 ± 0.02 b	0.29 ± 0.01 a	-9.67	25.28
FA 20:0	4.05 ± 0.56 a	3.95 ± 0.15 a	3.34 ± 0.18 b	4.45 ± 0.27 a	-2.32	33.37
FA 20:1	0.46 ± 0.04 a	0.35 ± 0.03 b	0.39 ± 0.01 b	0.43 ± 0.02 a	-24.24	9.34
FA 20:2	0.37 ± 0.02 a	0.31 ± 0.04 a	0.36 ± 0.02 b	0.45 ± 0.01 a	-17.07	23.77
FA 22:0	2.90 ± 0.18 a	2.78 ± 0.22 a	2.84 ± 0.15 b	3.94 ± 0.06 a	-3.99	38.93
FA 22:1	0.07 ± 0.001 a	0.05 ± 0.01 b	0.07 ± 0.01 a	0.07 ± 0.03 a	-22.58	6.65
FA 22:2	0.01 ± 0.001 a	0.01 ± 0.001 a	0.01 ± 0.001 a	0.02 ± 0.002 a	-23.75	9.96
FA 23:0	2.05 ± 0.13 a	2.01 ± 0.16 a	1.84 ± 0.07 b	2.51 ± 0.10 a	-1.98	36.77
FA 24:0	9.62 ± 0.74 a	8.93 ± 0.97 ab	8.12 ± 0.15 b	10.60 ± 0.36 a	-7.17	30.63
TFA	377.85 ± 21.73 a	260.47 ± 9.87 b	240.30 ± 19.19 b	276.66 ± 2.19 a	-31.07	15.13
UFA	250.63 ± 29.54 a	161.53 ± 5.35 b	136.84 ± 10.43 a	138.47 ± 6.22 a	-35.55	1.19
SFA	127.22 ± 8.71 a	98.94 ± 8.56b	103.46 ± 8.75 b	138.19 ± 8.21 a	-22.23	33.57
UFA/SFA	1.97 ± 0.39 a	1.63 ± 0.14 a	1.32 ± 0.01 a	1.00 ± 0.10 b	-17.26	-24.24

### Construction of a lipid metabolism regulatory network for cold tolerance in rice

**Fig. 9:**
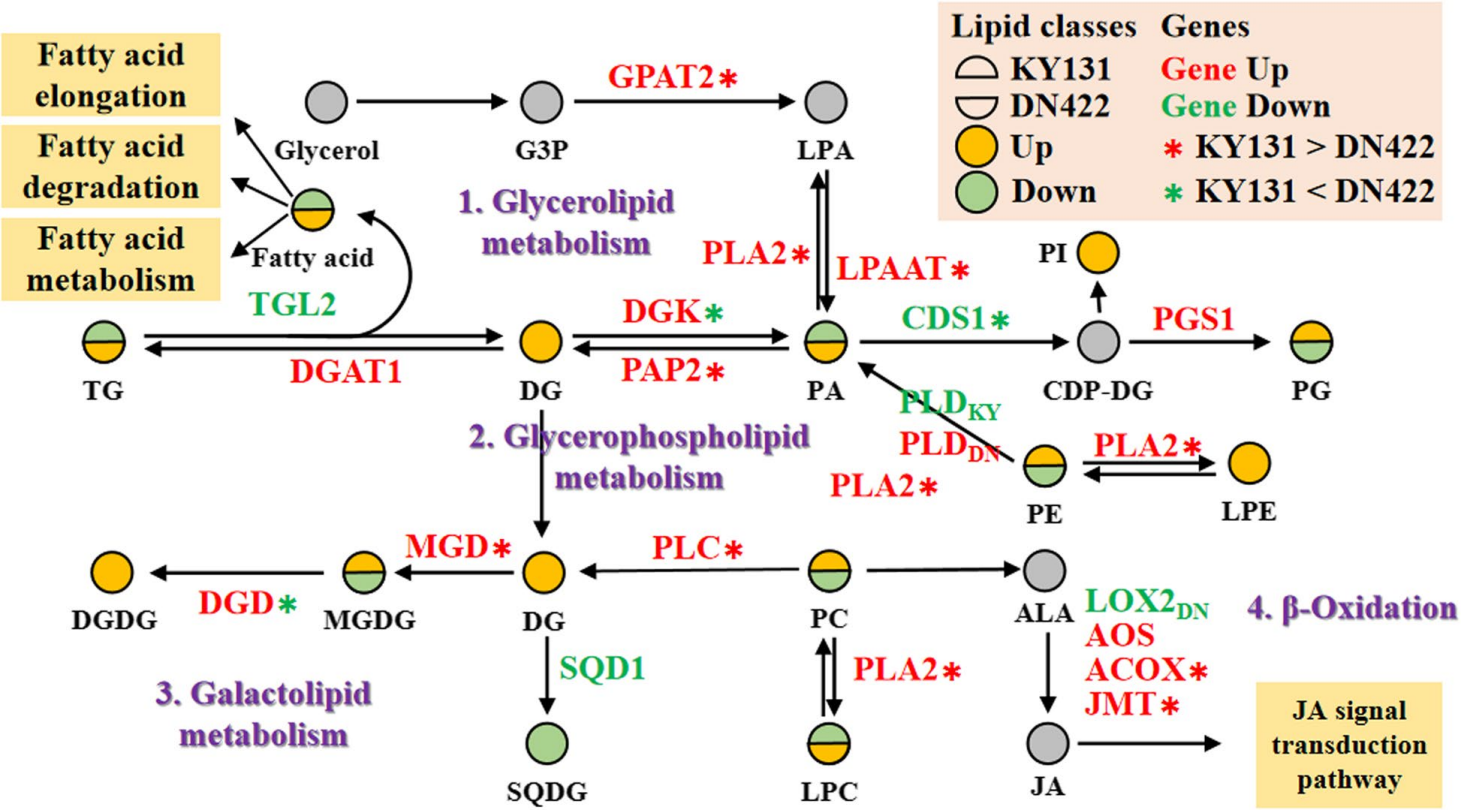
Cold exposure induces gene programs involved in lipid metabolism. Selected glycerolipid, glycerophospholipid, Galactolipid metabolism, and β-oxidation metabolic reactions from KEGG [28, 57], with indications of quantified lipid classes and acyl chains (circles) and genes (gene ID) significantly regulated in rice under cold conditions. Colors indicate up (red) and down (green) expression of genes; and increased (yellow), decreased (green), and undetected (gray) levels of the total concentration of the lipid classes. *indicate FC(KY131/DN422) ≥ 1.5

Combined transcriptome and lipid metabolome analysis results, a schematic diagram is presented to illustrate the lipid-related gene-metabolism network in rice adaptation to cold stress, and to elucidate the molecular regulation mechanism of membrane lipid metabolism in rice cold tolerance. As shown in Fig. 9, under cold stress, the up-regulated expression of CDS1 and PAP2 in rice leaves can hydrolyze PA to produce lipid intermediates DG and CDP-DG, which can prevent plants from excessive accumulation of PA and cause membrane lipid peroxidation damage. At the same time, the up-regulated expression of of MGD and GLA can directly lead to the increase of MGDG content, significantly increases the unsaturation of membrane lipids, which is crucial to ensure the structural integrity of chloroplast and maintain the normal process of photosynthesis under cold stress. Interestingly, the genes involved in fatty acid β-oxidation were all significantly up-regulated, which prevent the formation of very long chain fatty acids and promote the production of unsaturated fatty acids such as C16 and C18 by fatty acyl-CoA, thereby increasing the unsaturation of membrane lipids and enhancing cold tolerance. The β-oxidation process of fatty acids is also a central step in the α-linolenic acid (C18:3) metabolic pathway. In addition to increasing the unsaturation of membrane lipids, C18:3 can also be used as a precursor to synthesize JA through the α-linolenic acid metabolic pathway. Under cold stress, the expression of key genes (AOS, ACOX, and JMT) on the α-linolenic acid metabolic pathway in KY131 was significantly up-regulated, indicating that low temperature stress promoted the synthesis of JA. Therefore, the JA signal transduction pathway may play an important role in cold tolerance in rice.

## Discussion

Cold stress seriously affects the growth, development, yield, and quality of crops, and is a key limiting factor for rice production at higher altitudes and colder agricultural regions [23]. Many molecular studies have been performed on reference rice cultivars, providing important insights into the response to stress, although variability leads to the notion that conclusions are not always translatable to other cultivars with agronomic relevance. Photosynthesis is highly sensitive to cold stress [24] and in this study, we observed that there are large differences between cold-sensitive (DN 422) and cold-tolerant (KY 131) varieties in response to cold stress [25, 26], and there are many differences in the physiology, gene expression, and lipid metabolite changes between the two rice varieties under cold conditions. The photosynthesis-related indicators of the two varieties decreased significantly, and the carbon metabolism pathways such as photosynthesis, starch, and sucrose metabolism, and chlorophyll synthesis and degradation were also significantly enriched under low temperature stress. This study speculated that the decrease in Chl content, Pn, and Fv/Fm was caused by the damaged photosynthetic membrane, which may reduce carbon assimilation products, and in turn, inhibit growth and development [27]. Therefore, it is of critical importance to elucidate the specific mechanisms and promote the genetic improvement of cold tolerance in rice cultivars. The integrated analysis of the rice transcriptome and lipidome allowed us to gain more insight into the lipid metabolism regulation mechanism in response to cold stress, which could be of great practical significance in regulating rice lipid metabolism to improve cold tolerance in rice.

At present, many genes, metabolic pathways, and signaling factors have been identified in some plants during cold stress [28–30]; however, a clear and comprehensive relationship between lipid metabolism and cold tolerance has not been established in rice. Recent studies suggest that carbohydrate metabolism, amino acid metabolism, plant-pathogen interaction, and plant hormone signal transduction pathways are necessary and all play a role in response to low temperature stress [31–33]. In this study, these responses were also significantly enriched in the two rice varieties under cold conditions. The results of the GO enrichment hierarchical analysis showed that the stability of the membrane system was significantly affected in both varieties under cold conditions. Most of the cold-stress responsive lipid metabolism genes likely have similar expression patterns in both varieties to cope with cold stress. These results show that the membrane lipid metabolism pathway is indispensable for rice to adapt to cold conditions. Several recent studies have also shown that membrane lipid remodeling can modulate lipid composition, fatty acyl group unsaturation, and membrane fluidity, which have been developed as a key strategy for plants to adapt to low temperature stress [34, 35].

This study analyzed the DEGs and differential metabolites in lipid metabolism pathways under cold conditions and showed that most of the reactions in the phospholipid synthesis pathway in the endoplasmic reticulum, the galactolipid synthesis pathway in the chloroplast, and the α-linolenic acid metabolic pathway were activated, and most of the genes that catalyzed these reaction steps were upregulated under cold conditions. In plant cells, PA is not only an intermediate product produced by glycerophospholipids, galactolipids, and storage lipid TG, but also an important signal molecule, called “lipid second messenger” which is mainly induced by various biological and abiotic stresses. When encountering abiotic stresses such as low temperature and drought, the content of PA in plants usually increases rapidly, playing the role of signal molecules [13]. However, recent studies have shown that PA, as a polar lipid, lacks a hydrophilic head. Under low temperature stress, excessive accumulation of PA leads to the production of active oxygen free radicals, which trigger membrane lipid peroxidation [36]. In this study, the PA content of the two varieties showed different fates. PA in rice varieties leaves accumulated through three pathways: acylation of *OsGPATs* and *OsLPAAT*, hydrolysis of *OsPLCs* and *OsPLDs*, and phosphorylation of *OsDGKs*. When PA accumulates, it is also catalyzed and hydrolyzed by *OsPAPs*, *OsPGS1*, and *OsCDS1/2* into DG and CDP-DAG, and glycerophospholipids are produced under the action of various phospholipid synthases such as PISD, PCYT1/2, and EPT1. The downregulation of *OsCDS1* (*LOC_Os02g03460*) and *OsPAP2* (*LOC_Os08g27030*) caused excessive accumulation of PA, and subsequently reduced the content of PC, PE, and PG in DN 422. PG is the only phospholipid present in the chloroplast membrane and almost all PGs in plants are distributed in the chloroplast. Although PG occupies a relatively low proportion of membrane lipids, it is the main factor that determines the phase change of membrane lipids and increases fatty acid saturation. Related studies have shown that PG can quickly accumulate unsaturated fatty acids or unsaturated phospholipid molecules when plants adapt to low temperature stress, which is one of the factors necessary for plants to maintain a certain cold tolerance [37, 38]. Therefore, the upregulated expression of *OsCDS1* and *OsPAP2* under low temperature stress can prevent peroxidation damage caused by excessive accumulation of PA, while rapidly increasing PG to maintain membrane lipid stability.

Galactolipids are the most abundant lipids in rice leaves, accounting for approximately 66% of the total lipid content. It mainly includes three components: MGDG, DGDG, and SQDG. In plants, almost all galactolipids are distributed in the thylakoids. Generally, when plants are exposed to low temperature, drought, and salt stress, the MGDG content in their bodies is significantly reduced [37, 39, 40]. Moellering et al. [41] showed that the decrease in MGDG content under low temperature stress is due to its degradation; that is, the abundant galactose residues in MGDG are transferred to other lipid receptors under the action of the glycosyl hydrolase SFR2 in the final production of oligogalactolipids and DAG. In cold-tolerant varieties, the significant increase in MGDG content may be due to the upregulated expression of *OsGLA (LOC_Os03g56070)*, especially the significant increase in MGDG 34:3 and 36:6 content, which not only increased the content of thylakoid membrane lipids but also was increased by reducing the unsaturation of the thylakoid membrane lipid, resulting in less exposure of the thylakoid membrane to cold stress, thereby maintaining a certain degree of photosynthesis. Concurrently, this study also found that another thylakoid membrane lipid component DGDG increased significantly, especially the content of polyunsaturated molecular species such as DGDG C36:6, C36:5-DGDG, and C34:3, which increased the most. To a certain extent, the fluidity and integrity of thylakoid membrane lipids are maintained, thereby ensuring normal photosynthesis. Further studies have found that the increase in DGDG content is mainly caused by the significant upregulation of *OsMGD* and *OsDGD1*. In Arabidopsis, the knockout of *DGD1* hinders the formation of membrane light-harvesting complex II (LHCII) and reduces the stability of PSI. The chlorophyll fluorescence lifetime was significantly shortened [42]. Therefore, the increase in DGDG content caused by the up-regulation of *OsMGD* and *OsDGD1* in rice leaves under low temperature stress is essential to ensure the structural integrity of chloroplasts and maintain the normal progress of photosynthesis.

In addition to membrane lipid metabolism, fatty acid metabolism also plays an important regulatory role in the adaptation of rice to low-temperature stress. Previous studies have shown that since short-chain fatty acids and unsaturated fatty acids have lower melting points, plants can appropriately shorten the length of the fatty acid carbon chain and increase the number of fatty acid double bonds to inhibit the phase transition of membrane lipids under low temperature stress, thereby maintaining cell membrane fluidity [43]. This study demonstrated that under low-temperature conditions, cold-tolerant varieties have higher membrane lipid unsaturation and unsaturated fatty acid content. This enables the plant to maintain a certain degree of stability and fluidity of the membrane system under cold stress. Fatty acid β-oxidation is the main form of fatty acid degradation in plants and is catalyzed by a multienzyme complex, including ACOX, MFP, and ACAA, to decrease the length of the carbon chain of fatty acids [44]. In the present study, *OsACOX1* (*LOC_Os03g53630*) and *OsACOX4* (*LOC_Os05g07090*) were significantly upregulated in both varieties and activated the β-oxidation pathway of fatty acids under cold stress. Fatty acid β-oxidation is the sole pathway for the metabolic breakdown of fatty acids that generate energy and carbon skeletons in plants and plays an important role in plant growth, development, and cellular homeostasis [45, 46]. Fatty acid β-oxidation is also the central component of α-linolenic acid (C18:3) metabolism. In the α-linolenic acid metabolic pathway, C18:3 produces 12-oxo-phytodienoic acid (OPDA) under the action of lipoxygenase (LOX), hydroperoxide dehydratase (AOS), and cetyl-CoA acyltransferase (fadA), and then produce JA through three fatty acid β-oxidation cycles. In *Arabidopsis thaliana*, JA can positively regulate the C-repeat-binding factors (CBF) pathway to increase the accumulation of cryoprotective compounds under low temperature stress and interacts with other plant hormones to regulate stomatal closure and maintain the normal progress of light action [47, 48]. These results indicate that rice may improve its tolerance through active JA metabolism and signal transduction when exposed to cold stress.

In this study, these results provide a holistic view of lipid metabolism-related genes and metabolism in response to cold stress. PA, MGDG, DGDG, and FA play important roles in maintaining membrane stability and mobility under cold conditions. Changes in the expression of lipid metabolism-related genes (*OsCDS1, OsPAP2, OsGLA OsMGD*, and *OsDGD1*) alter the rice PA, MGDG, DGDG, and FA content to adapt to cold stress.

## Materials and methods

### Plant material, cold treatment, and sample preparation

The experiment was conducted at the Rice Research Institute of Northeast Agricultural University in 2020. Kongyu 131 (KY 131), a cold-tolerant rice cultivar, and Dongnong 422 (DN 422), a cold-sensitive rice cultivar, were selected. Rice seeds of the same size, plump, and vigorous were selected and sterilized with 1% sodium hypochlorite solution for 30 min, soaked in clean water at room temperature for 24 h, and then the paper bed sprouting method was used to cultivate them in the dark at 32 °C for 48 h. Well-germinated seeds were selected and sown in high-temperature sterilized sand and cultivated in an artificial climate room. During this period, 1/2 Hoagland nutrient solution was rationed daily. The culture conditions in the control temperature climate chamber were 25 °C, 10 h day/14 h night. At the three-leaf heart stage, the seedlings were transferred to the control temperature and low temperature climate room, and the culture conditions in the low-temperature climate chamber were 10 °C, 10 h day/14 h night. The cold treatment method was slightly modified according to a previous study [26]. Sampling was carried out after nine days of control (CK) and cold (C9) treatments. CKKY and CKDN represent KY131 and DN422 under control conditions; C9KY and C9DN represent KY131 and DN422 under cold treatment conditions. Three rice plants with the same growth were selected, and the rice leaves were cut with sterilized scissors, wrapped in foil paper, marked and placed in liquid nitrogen immediately, and then placed at -80 °C in a refrigerator for later use.

### Measurement of leaf relative water content, electrolyte leakage, malondialdehyde content, and photosynthetic characteristics

The leaves were harvested after nine days of treatment. The relative water content of the leaves was determined as described by Wang et al. [49]. Leaf electrolyte leakage was determined using an electrical conductivity meter (DDSJ-308 F, Shanghai, China) according to the method described by Song et al. [50]. Malondialdehyde content was measured as described by Chen et al. [54]. Nine days after the low-temperature treatment started, the net photosynthetic rate (Pn) was measured on the penultimate leaves using a CIRAS-3 portable photosynthetic instrument (PP Systems, USA) [52]. The fluorescence parameters Fv/Fm and chlorophyll (Chl) fluorescence images of the penultimate leaves were analyzed after 30 min of dark adaptation using fluorescence measurements with a HITACHI F-7000 fluorescence spectrophotometer (Tokyo, Japan) [53]. Chlorophyll (Chl) content was analyzed as previously described by Li et al. [54].

#### RNA extraction and transcriptome sequencing analysis

Three samples of rice from the penultimate leaf under the control and low temperature treatment were measured for nine days; three biological replicates per sample were used for the transcriptome analyses. Total RNA was extracted using TRIzol reagent (Invitrogen, Carlsbad, CA, USA). After total RNA was extracted, mRNA was purified using NucleoSpin RNA Clean-up (MACHEREYNAGEL, Düren, Germany) according to the manufacturer’s instructions. The purified mRNA was fragmented into short fragments using fragmentation buffer and reverse-transcribed into cDNA using random hexamer primers. Second-strand cDNA was synthesized using DNA polymerase I, RNase H, dNTPs, and buffer. The cDNA fragments were purified using a QiaQuick PCR extraction kit, end repaired, poly(A) was added, and the fragments were ligated to Illumina sequencing adapters. The ligation products were size-selected by 1% agarose gel electrophoresis, PCR-amplified, and sequenced using an Illumina HiSeqTM 2500 (Wuhan, China). The transcripts were assembled using Cufflinks to obtain the known and new transcripts. Before data analysis, we conducted strict quality control on the data to ensure that these reads were of high enough quality to ensure the accuracy of subsequent analysis. The filtering criteria were as follows: (1) Remove reads with adapters; (2) When the N content in any sequencing read exceeds 10% of the base number of the read, remove the paired reads; (3) When the number of low-quality (Q ≤ 20) bases contained in any sequencing read exceeded 50% of the bases of the read, the paired reads were removed. Then, differential expression analysis across samples was conducted to obtain DEGs (false discovery rate [FDR] < 0.05, |log2FC| > 1). Genes were annotated against the Gene Ontology (GO) (Available online: http://www.r-project.org/) and Kyoto Encyclopedia of Genes and Genomes (KEGG) (Available online: http://kobas.cbi.pku.edu.cn/) databases to obtain their functions. Significant functional categories and metabolic pathways were identified within differentially expressed genes, with FDR ≤ 0.05.

### Confirmation of lipid metabolism-related genes in the transcriptome using qRT-PCR analysis

The purified total RNA was reverse-transcribed using Prime Script RT Master Mix (Takara Bio Inc., Shiga, Japan). Real-time PCR was performed using SYBR Premix Ex Taq II (Takara Bio Inc., Shiga, Japan) in the Applied Biosystems QuantStudio 3 (Thermo Fisher Scientific, Germany). *OsACTIN1* was used as a reference control. Relative quantification analysis was performed using the comparative CT method. Correlation coefficients for RNA-Seq and qRT-PCR data were plotted and calculated using the Origin 2018 (OriginLab, Northampton, MA, USA) mapping software. The primers used are listed in Table S9.

### Lipid extraction and liquid chromatography electrospray ionization tandem mass spectrometry (LC-ESI-MS/MS) analysis

The cut rice leaf samples of approximately 250 mg were added with 80 µL of whole fat internal standard (both at a concentration of 10 µg/mL), and 2 mL of methanol. Then 2 mL of dichloromethane was added, and vortexed for 1 h. This was followed by the addition of 2 mL of dichloromethane, 1.6 mL of ultrapure water, vortexing, and centrifugation, removing the supernatant, adding 4 ml of dichloromethane to extract the remaining supernatant, repeating twice, combining the supernatants, blowing with nitrogen, and reconstituting with 1 mL of isopropanol. Then passed through the 0.22 μm organic filter membrane and get on the machine. Lipidomic analysis was performed on an AB Sciex Triple 4000 quadruple electrospray ionization mass spectrometer (ESI/MS) coupled with a Shimadzu UPLC LC-20 A (Shimadzu, Kyoto, Japan). The specific methods were previously described by Gwak et al. [55]. Based on the high-resolution QTOF mass spectrum, the precise molecular weight of the characteristic peaks in the sample was obtained and compared with the database information to obtain the qualitative results of the metabolites. The quantitative results of metabolites were obtained based on the ion signal intensity (CPS) of the characteristic peak in the mass spectrum. MSDIAL ver. 4.00, PeakView 2.1, MultiQuant 3.0.2, and other software were used to complete the qualitative and quantitative lipids.

### Statistical analysis

For experimental variables, one-way of variance (ANOVA) was applied to assess differences among treatments with SPSS 22.0 (Softonic International, Barcelona, Spain) software. Significant differences (*p* < 0.05) between treatments are indicated by different letters according to Fisher’s LSD. Principal component analysis (PCA) [56] and graphs were employed with Origin 2018 software (OriginLab, Northampton, MA, USA).

## Supplementary Information

**Additional file 1. **Differentially expressed genes (DEGs) in KY 131 under cold stress.

**Additional file 2. **Differentially expressed genes (DEGs) in DN 422 under cold stress.

**Additional file 3. **Go enrichment analysis of differentially expressed genes (DEGs) in KY 131 under cold stress.

**Additional file 4. **Go enrichment analysis of differentially expressed genes (DEGs) in DN 422 under cold stress.

**Additional file 5. **KEGG enrichment analysis of differentially expressed genes (DEGs) in KY 131 under cold stress.

**Additional file 6. **KEGG enrichment analysis of differentially expressed genes (DEGs) in DN 422 under cold stress.

**Additional file 7. **The response of the MAPK signaling pathway of two varieties to cold stress.

**Additional file 8. **Quantitative changes in lipid composition induced by cold stress.

**Additional file 9. **Sequences of primers used in this study.

## Data Availability

The datasets generated for this study can be found in the SRA accession number: SRR15076606, SRR15076607, SRR15076608, SRR15076609, SRR150766011, SRR150766012, SRR15076619, SRR15076620, SRR15076599, SRR15076600, SRR15076601, SRR15076602.
